# Analytical, Preparative, and Industrial-Scale Separation of Substances by Methods of Countercurrent Liquid-Liquid Chromatography

**DOI:** 10.3390/molecules25246020

**Published:** 2020-12-18

**Authors:** Artak A. Kostanyan, Andrey A. Voshkin, Vera V. Belova

**Affiliations:** Kurnakov Institute of General and Inorganic Chemistry, Russian Academy of Sciences, 31 Leninskii pr., 119991 Moscow, Russia; kost@igic.ras.ru (A.A.K.); belova@igic.ras.ru (V.V.B.)

**Keywords:** countercurrent chromatography, continuous and periodic separations, analytical, preparative and industrial scale separations, simultaneous separation and concentration, design and modeling

## Abstract

Countercurrent liquid-liquid chromatographic techniques (CCC), similar to solvent extraction, are based on the different distribution of compounds between two immiscible liquids and have been most widely used in natural product separations. Due to its high load capacity, low solvent consumption, the diversity of separation methods, and easy scale-up, CCC provides an attractive tool to obtain pure compounds in the analytical, preparative, and industrial-scale separations. This review focuses on the steady-state and non-steady-state CCC separations ranging from conventional CCC to more novel methods such as different modifications of dual mode, closed-loop recycling, and closed-loop recycling dual modes. The design and modeling of various embodiments of CCC separation processes have been described.

## 1. Introduction

Separation processes largely determine the purity of final products in the production of organic and inorganic substances. The leading trends in the modern technology development of extraction and separation of strategically important rare earth and associated metals are the creation of new extraction systems with more efficient extraction capacity [1,2,3,4,5,6,7,8] and separation methods that meet modern requirements to the organization of processes. Chromatographic separation methods are widely used for the isolation and purification of natural compounds for further analysis and testing of their biological activities. At present, an urgent problem is the production of pure and ultrapure organic and inorganic materials. To address this issue, new, highly efficient separation and purification methods are being developed. Countercurrent chromatography (CCC) [9,10,11,12,13,14,15,16,17,18,19,20,21] is a separation technology that due to the combination of the features of liquid–liquid extraction and partition chromatography [22,23,24,25,26] offers a variety of operating modes and allows for high adaptability to different separation tasks. These operating modes, many of which can be implemented with minor changes of the equipment used in elution, make it possible to significantly improve the separation performance compared to the conventional isocratic batch injection mode. The selection of the operating modes depends on the specific separation task and the available chromatographic equipment.

In CCC separations, to hold the stationary phase in a column, centrifugal chromatographs of various designs have been developed and tested [9,10,11,12,13,26,27,28,29,30,31,32,33,34,35,36,37,38,39,40,41,42,43,44]. Two types of chromatographs have found practical application: (1) hydrostatic devices, commonly known as centrifugal partition chromatography (CPC), using a constant-gravity field and two rotary-seal joints for inlet and outlet of the mobile phase—a cascade of chambers connected in series by ducts, is placed in a conventional centrifuge [26,27,28,29,30,31,32,40,41]; (2) hydrodynamic devices using a variable-gravity field—a coil column is wound in one or several layers onto the drum of a planetary centrifuge [9,10,11,12,13,32,33,34,35,36,37,38,42,43]. In the synchronous coil planet centrifuge (the type-J), the drum rotates around the centrifuge’s central axis and around its own axis at the same angular velocity and in the same direction; the lighter mobile phase (upper phase, the phase with lower density) is pumped from the tail toward the head (normal-phase mode) and the heavier (lower phase, the phase with higher density) mobile phase is pumped from the head toward the tail of the column (reversed-phase mode).

The conventional liquid-liquid chromatography systems employ solid supporting matrixes to retain the stationary phase. They provide very high separation efficiency, which is measured by thousands of theoretical plates. Their shortcomings arise from the solid support in the form of irreversible adsorption, decomposition, and sample contamination. CCC is a continuous liquid–liquid partition chromatography without solid matrixes. The support-free liquid stationary phase is retained by gravity or centrifugal forces. Organic-aqueous biphasic solvent systems consisting of three or more solvents with various volume ratios are most widely used in CCC separations [45,46,47,48]. The mobile and stationary phases are the pre-equilibrated phases of these two-phase solvent systems. The composition of the solvent systems is being selected, tailoring it to the sample and separation task.

The CCC methods are the most widely used in the field of pharmaceutical and natural product analytical and preparative-scale separations (alkaloids, peptides, drugs, chiral compounds, etc.) [17,27,28,29,30,38,47,48,49,50,51,52,53,54,55,56,57,58,59,60,61,62,63,64,65,66,67,68,69,70,71,72,73,74,75,76,77,78,79,80,81,82,83,84,85,86,87,88,89,90,91,92,93,94,95,96,97]. Countercurrent chromatography techniques allow the analysis of trace components in complex natural product extracts and an enrichment of these bioactive plant metabolites under non-destructive conditions [98,99]. Unlike liquid-solid chromatography separation processes, the sample molecules in CCC separations are not subjected to any sorption processes. Therefore, the original physicochemical properties of the sample molecules, such as bioactivity, are preserved. Slow velocity spinning countercurrent chromatography is being used for the activity-guided isolation of bioactive molecules from natural product extracts [100].

Although the large retention volume of the stationary phase in CCC columns allows injections of large amounts of samples, it is not sufficient for industrial scale separations. In CCC, similar to most chromatography applications, the volume of the column is the limiting factor in the performance. The complexity of centrifugal chromatographs imposes restrictions on their scale. At the Kurnakov Institute of General and Inorganic Chemistry, Russian Academy of Sciences, on the basis of currently available solvent extraction equipment (a cascade of multistage columns and a cascade of centrifugal mixer-settler extractors), high-performance CCC plants for industrial-scale separations are being developed [101,102,103,104,105,106,107,108].

For the simulation and optimal design of different operating modes of countercurrent chromatography, an appropriate theory is needed. The use of an experimental trial-and-error approach to determine the optimal operating mode for a given separation task is time consuming. For practical implementation of CCC separation processes, preliminary mathematical modeling is necessary. Mathematical modeling offers a time-saving alternative approach. The mathematical description of the CCC separations is less complex compared to the other forms of chromatography due to the lack of the packing materials, which makes it possible to find analytical solutions for the equations of mathematical models with a linear dependence of the equilibrium concentrations in the phases. The migration and spreading of chromatographic peaks in a CCC column depend only on the rate of interphase mass transfer, the degree of axial mixing in the phases, and the value of the partition coefficients. The combined effect of the axial mixing and the mass transfer on a CCC separation can be described on the basis of equilibrium or non-equilibrium cell models [22,23,24,25,26]. The non-equilibrium model takes into account the finite mass transfer rate, but the analytical solutions of the model equations have a rather complicated form. By comparing distribution functions of both models, it was shown [24] that instead of complicated solutions of the non-equilibrium model, much simpler solutions of the equilibrium model can be applied to describe real non-equilibrium processes using the following relationship between the model’s parameters:

(1) $$\frac{1}{N_{eff}}=\frac{1}{n}+\frac{2}{T}{\left(\frac{K^{\prime }}{1+K^{\prime }}\right)}^{2}$$

where *N_eff_* is the effective number of theoretical stages; n is the number of perfectly mixed cells in a column (the axial mixing parameter); *T = k_v_V_c_/F* is the number of mass transfer units in the column (the mass transfer parameter): *F* is the volumetric flow rate of the mobile phase, *k_v_* is the mass transfer coefficient related to the volume of contacting liquids, *V_c_* is the column volume; *K’ = K_D_S /(1−S)* is the ratio of amounts of a solute in the stationary and mobile phases under the equilibrium conditions (the retention factor): K_D_ = y*/x* is the partition coefficient of the solute; y* and x* are the equilibrium solute concentrations in stationary and mobile phases, respectively; and *S* is the fractional volume of the stationary phase in the column.

Replacing the number of equilibrium cells (theoretical plates, *N*) in equilibrium model equations by the effective number of theoretical *N_eff_* stages according to formula (1) makes it possible to proceed from a simpler equilibrium model in the analysis of CCC separations.

In this review, we will focus on an overview of the different modifications of dual mode and closed-loop recycling CCC and closed-loop recycling dual-mode CCC and their corresponding models to design analytical, preparative, and industrial-scale separations. It should be emphasized that these highly efficient operating modes cannot be successfully implemented in practice without preliminary mathematical modeling. Therefore, in this review, we present the equations and examples of modeling these operating modes, which can help users, including non-expert ones, to select the best-suited operating mode and process parameters for a certain separation task.

## 2. Analytical Scale Separations

The high volume fraction of stationary phase in CCC devices enhances the resolution; however, the separation power of CCC is much lower than that of HPLC. The efficiency of CCC separations can be greatly improved by applying operating schemes and elution modes simulating the lengthening of the column, such as dual (DM) and multiple dual modes (MDM) [109,110,111,112,113,114,115,116,117,118,119,120], closed-loop recycling (CLR) [121,122,123,124,125,126,127,128,129] CCC, and their combinations. In this section, we look at these techniques for the separation of substances for analytical purposes. A distinctive feature of analytical scale separations is non-steady-state process mode where the sample is injected into the column using sample loops.

### 2.1. Multiple Dual-Mode CCC Separations

In multiple dual mode CCC separations (MDM CCC), the processes consists of a succession of two isocratic countercurrent steps carried out in series alternating between heavy phase flow (the heavy phase is pumped through the stationary light phase) and light phase flow (the light phase is pumped through the stationary heavy phase); each phase elutes alternately through the opposite ends of the column. The wide variety of MDM CCC operating modes differ only in sample loading conditions: (1) the single sample is introduced at the beginning or into the middle section of the column or between two-columns connected in series; (2) the separation is carried out with periodic sample re-injection; (3) the sample is introduced during a certain time; (4) the sample is fed continuously and at a constant rate into the middle section of the column, etc. The scope of this review is limited to MDM CCC operating modes in which the sample is introduced into the mobile phase at the inlet to the column once or periodically between each of the dual-mode steps. These operating modes are the simplest and most convenient for practical implementation. For a more detailed explanation of the other MDM CCC operating modes, the recently published review articles [99,130] can be recommended.

### 2.2. Multiple Dual-Mode CCC Separations with Variable Duration of Phase Elution Steps

**Figure 1:**
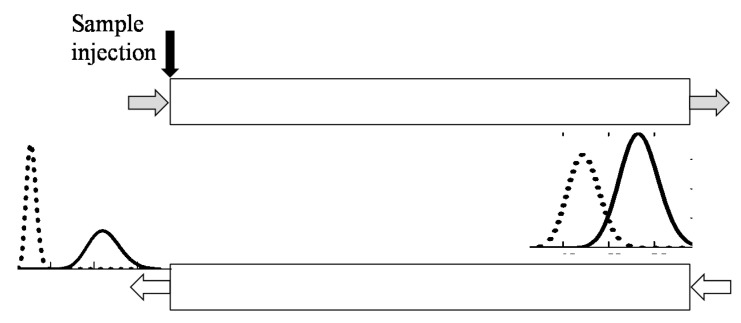
Schematic diagram of the multiple dual mode (MDM) countercurrent (CCC) separation with variable duration of phase elution steps: all the solutes are completely eluting with one phase in a certain cycle.

As mentioned above, MDM CCC separations consist of a succession of countercurrent dual-mode cycles, each of which comprises two isocratic steps (Figure 1): the first step—the heavy (or the light) phase pumped as the mobile phase; the second step—the light (or the heavy) phase pumped as the mobile phase.

The sample is injected into the mobile phase at the inlet to the column within the first step. The phase flow is repeatedly switched back and forth to retain the solutes inside the column until the desired separation is achieved. The shuttle forward and backward movement of the sample increases its retention in the column, which increases the number of theoretical plates and increases the efficiency of the separation. Therefore, it is desirable to use the full length of the column in each cycle to increase the path length of the components. During the movement of the sample in the column, the peaks of the solutes become broader along with the separation; therefore, it is necessary to reduce cycle times during the transition from one cycle to the next one to retain the sample in the column for a specified number of cycles.

Let us consider two methods of sample injection: (1) the single sample is injected within the first step of the first cycle; (2) the sample is injected within the first step of each cycle.

### 2.3. Modeling of Multiple Dual-Mode Countercurrent Chromatography Separations with Variable Duration of Phase Elution Steps and Single Sample Injection

**Figure 2:**
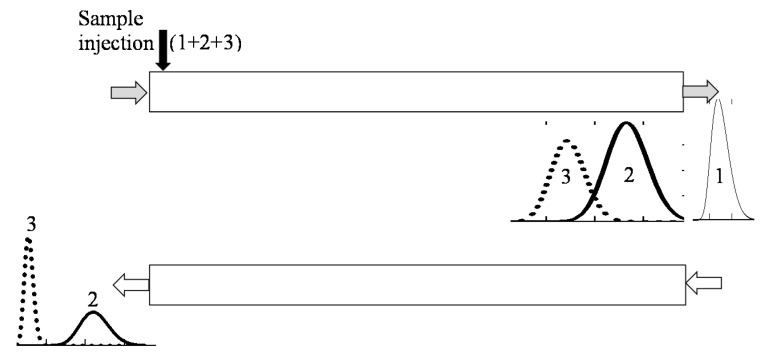
Schematic diagram of the MDM CCC separation with variable duration of phase elution steps: individual solutes are completely removed from the column with different phase flows.

Various schemes of MDM CCC with variable duration of phase elution steps can be implemented to separate binary and complex mixtures. Figure 1 illustrates the case, when all the solutes are completely eluting with one phase in a certain cycle. The case, when individual solutes are completely removed from the column with different phase flows is illustrated in Figure 2. Separation schemes may also be performed, in which the individual solutes are removed from the column in portions in over several steps and cycles. Here, the key point is timely switching of the phase flows to ensure the required purity of the collected fractions of individual solutes.

To simulate various options of the MDM CCC separations with single sample injection and variable duration of phase elution steps equations were developed in [131]. These equations describe concentration profiles in the column after both steps for any cycle and the chromatograms of solutes eluted from the column with both phases during each step and cycle. Based on these equations, a computational program in the form of a calculator for numerical simulation was developed. The program can be found in [131].

### 2.4. Modeling of Multiple Dual-Mode Countercurrent Chromatography Separations with Variable Duration of Phase Elution Steps and Multiple Sample Injection

The application of multiple sample injection offers several options for the isolation and enrichment of the fraction of the target components from complex mixtures. This technique makes it possible to overcome the difficulties associated with the analyses of minor components in complex mixtures, when it is necessary to determine substances down to an exceedingly low level of their content in the sample, and the separation of these substances in a concentrated form is required.

**Figure 3:**
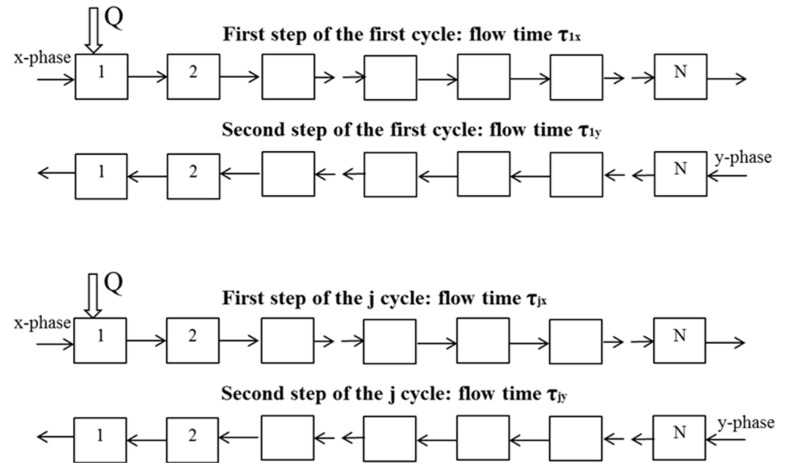
Schematic diagram of the mathematical model of multiple dual mode countercurrent chromatography with variable duration of phase elution steps and periodic sample injection.

The mathematical model of the MDM CCC separations with variable duration of phase elution steps and periodic sample injection has been developed in [132]. Basing on the cell model, the following propositions of the theory were postulated: the MDM CCC separation process consists of a succession of two isocratic steps: first, the “x” phase pumped as the mobile phase, and second, the “y” phase pumped as the mobile phase; the start time for every step of a cycle is 0; at the beginning of the first step of every cycle, a constant amount *Q* of a solute is injected into the column. Equations were presented to simulate separations where the timing of the alternating phase elution steps can be adjusted. Basing on this mathematical model (Figure 3), a computational program (the calculating machine) was developed that allows the simulation of the chromatograms and the calculation of the amounts of solutes eluted from the column with both phases during each step and cycle [133]. Using the calculating machine, processes for the separation of mixtures containing up to five solutes can be simulated and designed. The calculating machine can be found in [133].

The MDM CCC separation processes with an adjustable duration of phase elution steps are very sensitive to the choice of the duration of alternating phase elution steps. The calculating machine provides a simple tool that allows selecting optimal experimental conditions for the MDM CCC separations under consideration.

### 2.5. Closed-Loop Recycling CCC Separations

Closed-loop recycling countercurrent chromatography (CLR CCC) is another way to simulate the lengthening of the chromatographic column: the sample is recycled several times over the column until the required separation is reached (the entire sample or certain parts of the elution profile can be recycled). Compared to the MDM CCC, the CLR CCC methods are easier to implement, require no additional equipment, and offer a good solvent economy.

**Figure 4:**
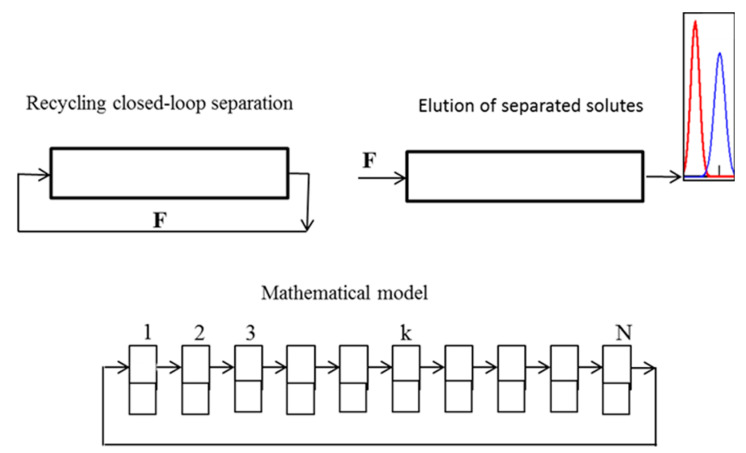
Scheme and principle of the ideal recycling mode of a CLR CCC separation and the applied mathematical model.

For the modeling and design of the CLR CCC separations, two approaches can be used [134,135]: the ideal recycling mode and the non-ideal recycling mode. The first approach (Figure 4) can be used when the volume of the recycling system (connecting lines, pump, detector, and valves) does not exceed one percent of the column volume. 

In this case, the effects of extra-column dispersion on the separation processes can be neglected [135]. The second approach takes into account the influence of the parameters of the recycling system (axial dispersion and volume) on the separation processes in a closed loop.

As above, we will consider two methods of sample injection: single and multiple sample injections.

### 2.6. Modeling and Design of Closed-Loop Recycling Countercurrent Chromatography Separations with Single Sample Injection

As noted above, there are two options for a CLR CCC installation: with a small volume of the recycling system compared to the column (with short connecting tubing) and with a certain volume of the recycling system (with long connecting tubing).

### 2.7. CLR CCC Separations Using Recycling Systems with a Short Recycling Line

The simplified operating scheme and the mathematical model of the separation in the ideal recycling mode are shown in Figure 4. The process is carried out as follows: after the sample injection into the mobile phase at the inlet to the column, the column outlet is directly connected to its inlet, and the sample is recycled several times over the column until the required separation is reached. After that, the loop is opened, the fresh mobile phase is pumped into the column, and separated solutes are eluted with the mobile phase.

**Figure 5:**
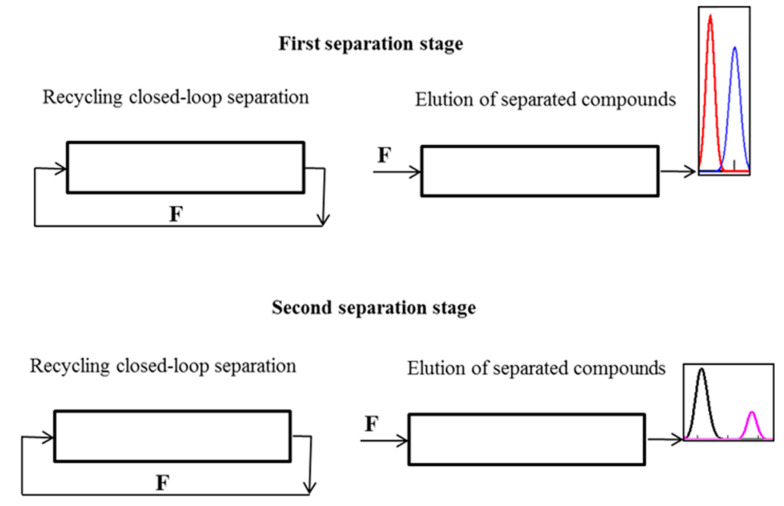
Two-stage CLR CCC separation of complex mixtures.

Figure 4 shows the operating scheme of a one-stage separation process, when no compound of the mixture to be separated is let out of the column until the desired separation is reached. It can be used to separate compounds with similar partition coefficients. For the separation of complex mixtures containing compounds with widely different partition coefficients, multistage schemes should be used. Figure 5 shows the operating scheme of a two-stage separation of complex mixtures.

The first separation stage comprises three operating steps: (1)—the sample injection into the mobile phase at the inlet to the column (the loop is open); (2)—the loop is closed, and in a definite number of cycles, the first separation is performed; (3)—the loop is opened, fresh mobile phase is pumped into the column, and the most fast or slow moving peaks are removed from the column. After the elution of separated compounds is finished, the loop is closed again, and the second separation stage starts. It is carried out in two steps: (1)—separation of the remaining unresolved compounds in recycling closed-loop; (2)—elution of the separated compounds with the mobile phase.

To simulate the above CLR CCC processes, the following equations can be used [134]:

(2) $$X(n,t)=\frac{a\sqrt{N}}{\sqrt{2\pi n}}\exp\left[-\frac{{\left(n-at\right)}^{2}}{2n}N\right]$$

(3) $$X_{n}(t)=\frac{a\sqrt{N}}{\sqrt{2\pi }}\sum_{i=1}^{n}\frac{1}{\sqrt{i}}\exp\left[-\frac{{\left(i-at\right)}^{2}}{2i}N\right]$$

(4) $$a=\frac{1}{1-S+SK_{D}}.$$

In Equations (2)–(4), $X=x/\bar{x}$ is the dimensionless concentration of a solute in the mobile phase; $\bar{x}{=Q/V}_{c}$ is the average concentration of the solute in the column after sample injection; *Q* is the amount of the solute in the sample injected; *t = τ (F/V_c_)* is the dimensionless time; *F* is the volumetric flow rate of the mobile phase; *V_c_* is the column volume; and *τ* is the actual time.

Equation (2) represents the peak equation for any current cycle *n* (*n* = 1, 2, 3...) without regard to the recycling process history; it does not take into account the interaction of peaks of adjacent cycles. Equation (3) describes the transformation of the elution profile of a solute during the entire recycling process from the first cycle to the cycle *n*.

### 2.8. CLR CCC Separations Using Recycling Systems with a Long Recycling Line

**Figure 6:**
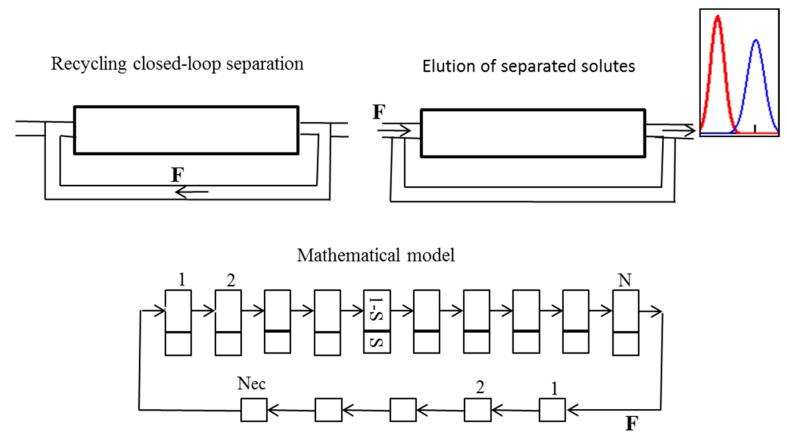
Operating scheme and principle of the non-ideal recycling mode of a CLR CCC separation and the applied mathematical model.

The simplified operating scheme and the mathematical model of the separation in the non-ideal recycling mode are shown in Figure 6. The separation process is carried out as described above in Section 2.7. The recycling system contains only the mobile phase, no chromatography is occurring in it; the band broadening is caused by the axial mixing, which can be characterized by the number of perfectly (ideally) mixed cells. To include the recycling system in the mathematical model of CLR CCC separations, the system of tubing, loop, pump, and valving that allows for the recycling of the mobile phase through the column is replaced by a cascade of ideally mixed cells with a degree of dispersion equivalent to the one caused by the real recycling system.

Thus, the non-ideal recycling equilibrium-cell model shown in Figure 6 takes into account both factors: the spreading of the injected solute in the chromatographic column—caused by the axial mixing and the mass transfer between the mobile and stationary phases—in the form of the number of theoretical stages *N* (equilibrium cells) and the extra-column dispersion in the recycling system—in the form of the number of perfectly mixed cells *N_ec_*. The model has two additional parameters: b = *V_ec_/V_c_*—the ratio of the column volume *V_c_* and the volume of the recycling system *V_ec_* and *N_ec_*—the number of perfectly mixed cells characterizing the dispersion in the recycling system. To simulate the non-ideal CLR CCC processes, the following equation can be used [135,136]:

(5) $$X_{n}(t)=\frac{aN\sqrt{N_{ec}}}{\sqrt{2\pi }}\sum_{i=1}^{n}\frac{\exp\left[-\frac{N_{ec}{\left[Ni+abN(i-1)-aNt\right]}^{2}}{2N_{ec}Ni+2(i-1){\left(Nab\right)}^{2}}\right]}{\sqrt{N_{ec}Ni+(i-1){\left(Nab\right)}^{2}}}.$$

Equation (5) describes the transformation of the elution profile of a solute during the entire recycling process from the first cycle to the cycle ***n*** for the non-ideal recycling mode of operation.

**Figure 7:**
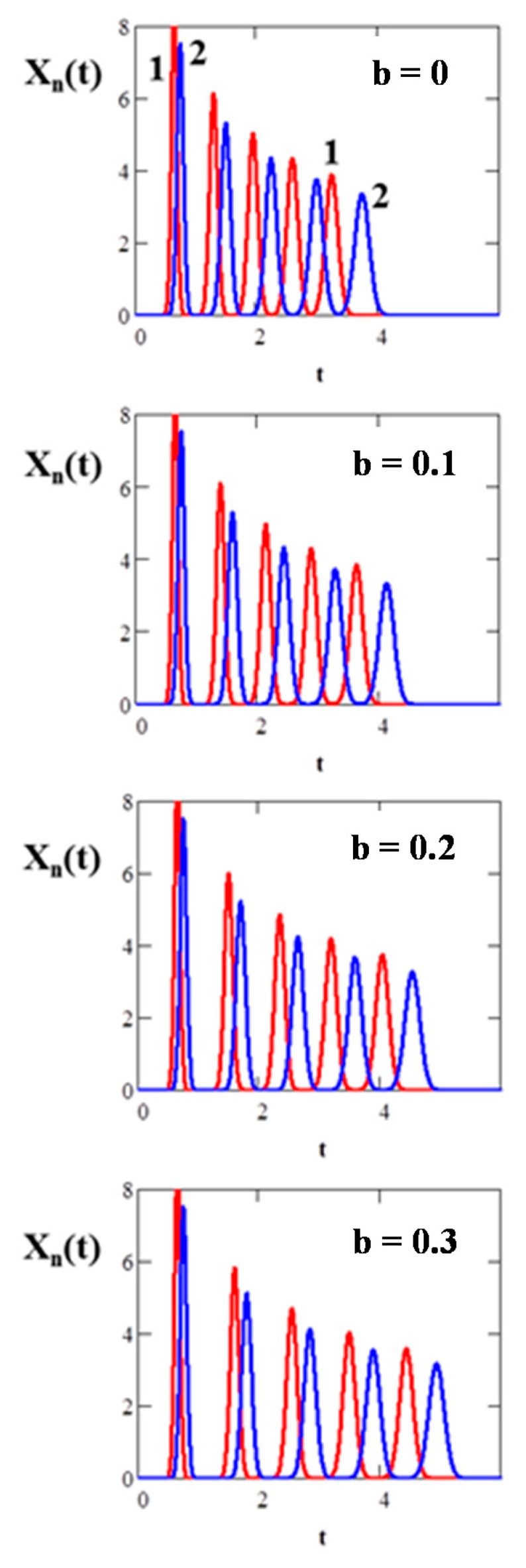
Simulation by Equation (5) of the closed-loop recycling countercurrent chromatography (CRL CCC) separations of the solutes *K_D1_* = 0.3 and *K_D2_* = 0.5 for different values of parameter b: *N* = 200, *N_ec_* = 200, *S* = 0.5.

The CLR CCC separations are determined by two counteracting phenomena during the process of sample recycling: as the number of cycles increases, the number of theoretical stages in the separation process increases, and the quality of the separation improves due to the repeated use of the column. However, after a certain number of cycles, chromatograms of neighboring cycles begin to overlap. In Figure 7, the simulation by Equation (5) of the CRL CCC separation of the binary mixture of the solutes with partition coefficients *K_D1_* = 0.3 and *K_D2_* = 0.5 is shown for different lengths (volumes) of the recycling system.

As seen, due to the time delay, the resolution between the chromatograms of the neighboring cycles is larger in the case of the non-ideal recycling mode with a long length recycling system, which makes it possible to increase the number of cycles (sample passages through the column) without overlapping of the peaks of neighboring cycles and hence, improve the separation. When *b* = 0 after the second cycle, the peaks of the neighboring cycles start to overlap, while when *b* = 0.3, this does not occur, which allows achieving acceptable separation after the fifth cycle. Thus, to improve the separation of the CRL CCC, recycling systems with long small diameter tubing are to be used.

Knowing the composition of the mixture to be separated and the parameters *b*, *K_D_*, *N*, *N_ec_*, and *S*, by using Equation (5), a given separation can be simulated, and the number of cycles and the periods of collection of fractions of solutes can be determined.

### 2.9. Modeling and Design of Closed-Loop Recycling Countercurrent Chromatography Separations with Multiple Sample Injection

**Figure 8:**
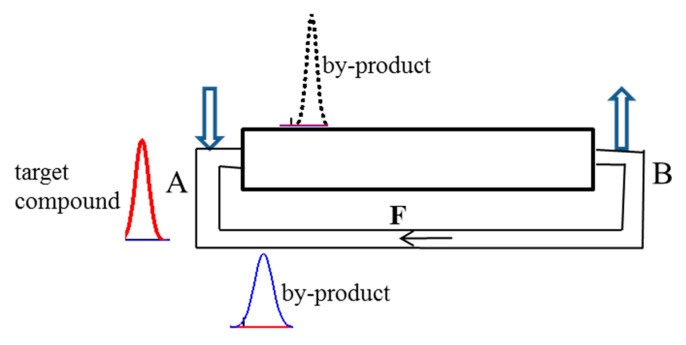
Schematic diagram of the closed-loop non-ideal recycling countercurrent chromatography with multiple sample injection.

The method of CLR CCC with multiple sample injection for the simultaneous separation and concentration of target components from mixtures was proposed in [137]. Based on the ideal recycling approach, equations were developed to describe these CLR CCC separations; different modes of multiple feed injection (the feed is injected in each cycle, after every two cycles, and in an arbitrary cycle) for the separation and concentration of a target component from binary mixtures were studied. In [138], the non-ideal recycling approach was used to develop equations, allowing the design and simulation of different variants of separation and concentration of target compounds from multicomponent mixtures by this method. The principle of the non-ideal recycling approach is schematically shown in Figure 8.

Recycling chromatogram equations at two points—the inlet of the column (A) and the outlet of the column (B)—are developed. The sample is repeatedly injected at the time when the peak of the target compound *K_Dt_* passes point A. These time points (*t_rt_*) are determined by the equations:

(6) $$t_{rt}=t_{Rt}(r)=\left(\frac{1}{a_{t}}+b\right)r$$

(7) $$a_{t}=\frac{1}{1-S+SK_{Dt}}$$

(8) $$N_{eft}=\frac{N_{t}N_{ec}}{N_{ec}+N_{t}a_{t}^{2}b^{2}}$$

where *N_t_* is the number of equilibrium stages in the column associated with the target compound; r is the number of the cycle (the number of passages of the target compound through the point A), after which the sample is re-injected; and *t_R_* is the position of the target compound peak on the time axis.

Elution profiles of the target compound at point B corresponding to individual sample injections are described by Equation (9):

(9) $$X_{n,r}(t)=\frac{a_{t}}{\sqrt{2\pi }}\sum_{i=1}^{n-r}\sqrt{\frac{N_{t}N_{eft}}{N_{eft}+N_{t}(i-1)}}\exp\left[-\frac{{\left(i+a_{t}b(i-1)+a_{t}(t_{rt}-t)\right)}^{2}}{2N_{eft}+2N_{t}(i-1)}N_{t}N_{eft}\right].$$

To achieve the required purity and concentration of the target compound, the optimum operating parameters of a given separation (number of sample injections and cycles, periods between successive injections, “cut times”, etc.) should be determined. For this purpose, when the sample is injected into the column after each cycle, the following equations can be used:

(10) $$X_{n,0}(t)=\frac{a}{\sqrt{2\pi }}\sum_{i=1}^{n}\sqrt{\frac{NN_{ef}}{N_{ef}+N(i-1)}}\exp\left[-\frac{{\left(i+ab(i-1)-at\right)}^{2}}{2N_{ef}+2N(i-1)}NN_{ef}\right]$$

(11) $$X_{n,1}(t)=\frac{a_{j}}{\sqrt{2\pi }}\sum_{i=1}^{n-1}\sqrt{\frac{N_{j}N_{efj}}{N_{efj}+N_{j}(i-1)}}\exp\left[-\frac{{\left(i+a_{j}b(i-1)+a_{j}(t_{rt_{1}}-t)\right)}^{2}}{2N_{efj}+2N_{j}(i-1)}N_{j}N_{efj}\right]$$

(12) $$t_{rt_{1}}=\frac{1}{a_{t}}+b$$

(13) $$N_{efj}=\frac{N_{j}N_{ec}}{N_{ec}+N_{j}a_{j}^{2}b^{2}}$$

(14) $$X_{n,2}(t)=\frac{a_{j}}{\sqrt{2\pi }}\sum_{i=1}^{n-2}\sqrt{\frac{N_{j}N_{efj}}{N_{efj}+N_{j}(i-1)}}\exp\left[-\frac{{\left(i+a_{j}b(i-1)+a_{j}(t_{rt_{2}}-t)\right)}^{2}}{2N_{efj}+2N_{j}(i-1)}N_{j}N_{efj}\right]$$

(15) $$t_{rt_{2}}=2\left(\frac{1}{a_{t}}+b\right).$$

Equation (10) describes the elution profile of a compound with the partition coefficient *K_D_* at the point B corresponding to the first sample injection at the time τ=0 (*t* = 0). Equations (11) and (14) describe the elution profiles of the compound *K_Dj_* (*j* = 1,2,3) corresponding to the second *X_n,1_* and third *X_n,2_* sample injections. Equations (11), (12), (14) and (15) can be obtained by putting r = 1 and r = 2 in Equations (6) and (9), respectively. The resulting concentration profiles after two and three sample injections are described by the equations:

(16) $$X_{m=2}(t)=X_{n,0}(t)+X_{n,1}(t)$$

(17) $$X_{m=3}(t)=X_{n,0}(t)+X_{n,1}(t)+X_{n,2}(n,t).$$

The subscript *m* in these equations denotes the total number of injections.

The application of multiple sample injection in the CLR CCC technique provides new opportunities to separate complex mixtures and concentrate target compounds. Examples of the separation of target compounds from three and five-component mixtures are presented in [138].

### 2.10. Closed-Loop Recycling Dual-Mode CCC Separations

A common feature of the closed-loop recycling and dual mode CCC separations is the virtual column elongation, which allows for the multiple increase of the separation power of chromatographic columns. Recently [136,139], the method of CCC separations was proposed, which incorporates the advantages of both methods: separations are carried out in a countercurrent closed-loop recycling mode, including the periods of alternating recirculation of light and heavy phases. Fractions of separated compounds are withdrawn from the column with the phases after the periods of their recycling. The new technique called closed-loop recycling multiple dual mode countercurrent chromatography (CLR MDM CCC) offers new opportunities to create more powerful separation processes. For practical implementation of these processes, preliminary mathematical modeling is necessary; however, the theory of this method has yet to be created. Currently, a simpler version of this method has been developed and investigated, when, after the separation in the closed loop recycling mode and the elution of the separated compounds with the mobile x-phase through the one end of a column, the phases are reversed, and the remaining compounds are eluted with the mobile y-phase through the opposite end of the column. This CCC separation method is called closed-loop recycling dual mode countercurrent chromatography (CLR DM CCC) [136,139].

### 2.11. Modeling and Design of Closed-Loop Recycling Dual Mode Countercurrent Chromatography Separations 

**Figure 9:**
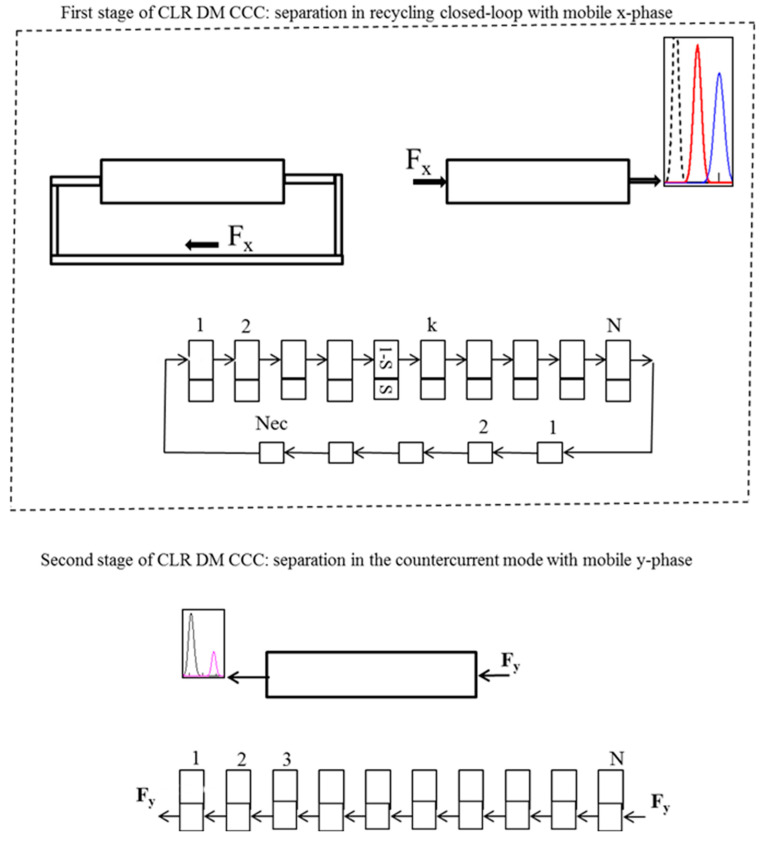
Schematic diagram of the closed-loop recycling dual mode countercurrent chromatography separations and the applied mathematical model.

The CLR DM CCC method consists of two successive separation stages (Figure 9): (1)—separation in the recycling closed-loop with mobile x-phase; (2)—separation in the countercurrent mode with mobile y-phase. As noted above, the elongation of the recycling line in CLR CCC significantly improves the separation, so furthermore, we will consider non-ideal recycling CCC.

To model the first separation stage, Equation (5) can be used. As mentioned above, after the elution of the certain separated compounds with the mobile x-phase, the phases are reversed, and the remaining compounds are eluted through the opposite end of the column with the mobile y-phase (Figure 9). This stage of the separation can be simulated by the equations [139]:

(18) $$Y(t)=K_{D}e^{-K_{D}aNt}\sum_{i=1}^{50}\frac{{\left(K_{D}aNt\right)}^{k-1}}{(k-1)!}X_{n}(k)+K_{D}\sum_{k=51}^{N}\frac{{\left(K_{D}aNt\right)}^{k-1}}{\left(k-1\right)}\frac{e^{k-1-K_{D}aNt}}{\sqrt{2\pi (k-1)}}X_{n}(k)$$

(19) $$X_{n}(k)=\frac{aN\sqrt{N_{ec}}}{\sqrt{2\pi }}\sum_{i=1}^{n}\frac{\exp\left[-\frac{N_{ec}{\left[N(i-1)(1+ab)+k-aNt_{x}\right]}^{2}}{2N_{ec}[N(i-1)+k]+2(i-1){\left(Nab\right)}^{2}}\right]}{\sqrt{N_{ec}[N(i-1)+k]+(i-1){\left(Nab\right)}^{2}}}$$

where *t* is the dimensionless time defined as *t* =*τF_y_/V_c_*; the start time for the y-phase flow is *τ* = 0 (*t* = 0); and $Y=y/\bar{x}$ is the normalized concentration of a compound eluting with the mobile y-phase.

Equation (19) describes the distribution of compounds in the column after the first separation stage, where the duration of *x*-phase circulation is *τ* *=* *τ_x_* (*t* = *t_x_*).

Equations (5), (18), and (19) are easy to use in any standard computer program to design and simulate various options of CLR DM CCC. In [136], several examples of the separation of closely related compounds with low and high partition coefficients and complex mixtures containing closely related compounds with low and high partition coefficients are discussed and presented in the “Mathcad” program.

## 3. Preparative and Industrial-Scale Separations

In preparative and industrial-scale separations, the goal is to maximize the volume of sample injected into the column. However, when the sample is injected using sample loops, an increase in the sample volume can lead to the stripping of stationary phase and violate the hydrodynamic conditions set in the column, which impairs the purity of the separated fractions of the compounds. The problem can be solved by replacing the procedure of the sample injection by sample loops by the continuous sample loading over a certain time [140]: The sample solution is continuously loaded into a CCC device over a definite time at the same rate as the pure mobile phase by switching the mobile phase pump from a tank with the mobile phase to a tank with the solution of the mixture to be separated in the mobile phase. After the sample solution loading is completed, the mobile phase is fed to the CCC device again at the same flow rate.

The general disadvantages of chromatographic techniques are the low throughput and complicated device setup. These disadvantages are particularly acute when industrial separations are desired. For example, process-scale extraction columns have throughputs two orders of magnitude higher (up to 50–100 m^3^/h) than process-scale CCC columns. The complexity of CCC devices imposes restrictions on their scale. For example, the current CCC equipment cannot process large volumes of feed material formed during the industrial production of rare earth metals. The high-performance CCC plants for industrial-scale separations are to be developed on the basis of the currently available solvent extraction equipment [101,102,103,104,105,106].

The preparative and industrial scale separations can be carried out in both steady-state and non-steady-state operating modes.

### 3.1. Modeling and Design of Non-Steady-State Preparative and Industrial Scale Countercurrent Chromatography Separations

Note that the models for preparative and industrial-scale separations have one additional parameter: the sample loading time *τ_s_ (t_s_).* To ensure high performance, it is necessary to load large volumes of the sample solution, which can be accomplished by increasing the loading time. Increasing the sample solution loading time from *t_s_* = 0.01 (impulse sample injection) to *t_s_* = 0.1 corresponds to a ten times increase in productivity.

### 3.2. Conventional Elution Mode

To predict the influence of the sample loading time on the separation and select a suitable compromise between the productivity and the resolution, the following equation can be used [140]:

(20) $$a=1,\;X(t)=\frac{1}{t_{s}}e^{-aN(t-t_{s})}\sum_{1}^{N}\left\{\frac{{\left[aN(t-t_{s})\right]}^{N-i+1}}{(N-i+1)!}\left[1-e^{-aNt_{s}}\sum_{1}^{i}\frac{{\left(aNt_{s}\right)}^{i-1}}{(i-1)!}\right]\right\}.$$

Equation (20) describes the elution profile of a compound *K_D_* after the sample, containing *Q* = *x_s_ F τ_s_* amount of the compound, which has been introduced into the column with a feed stream (*x_s_* is the compound concentration in the feed stream; *τ_s_* is the sample loading time).

Equation (20) is rather complicated: when the sample solution is loaded over a time, not exceeding 20–30% of the mean residence time $\bar{\tau }{=V}_{c}/F$ (*t_s_* ≤ 0.2–0.3), the much simpler Equation (21) can be applied to describe the conventional CCC separations [141].

(21) $$X(t)=\frac{1}{\sqrt{\frac{2\pi }{Na^{2}}+\frac{\pi t_{s}^{2}}{6}}}\exp\left[-\frac{{\left(\frac{1}{a}+\frac{t_{s}}{2}-t\right)}^{2}}{\frac{2}{Na^{2}}+\frac{t_{s}^{2}}{6}}\right]$$

It should be recalled that Equations (20) and (21) are expressed in normalized form by using the average concentration of the compound in the column and the mean residence time (the mean time for of the compound *K_D_* =1):

(22) $$\bar{x}=Q/V_{c}=x_{s}F\tau_{s}/V_{c}\;t=\tau /\bar{\tau }=\tau F/V_{c}\;t_{s}=\tau_{s}F/V_{c}$$

### 3.3. Multiple Dual-Mode CCC Separations with Variable Duration of Phase Elution Steps

#### 3.3.1. Single Sample Loading

The MDM CCC separations are carried out as described above in Section 2.3. The only difference is in sample loading conditions: instead of a pulse injection, the sample solution is introduced into a column during a certain time. The equations and the computer program for the simulation of these separation processes can be found in the paper [131].

#### 3.3.2. Multiple Samples Loading

The MDM CCC separations are carried out as described above in Section 2.4. The equations and the computer program for simulations can be found in the paper [133].

#### 3.3.3. Closed-Loop Recycling CCC Separations

As shown above, the elongation of the recycling line in CLR CCC can improve the separation; therefore, hereinafter, we will consider non-ideal recycling CCC. The preparative and industrial scale closed-loop recycling CCC separations are carried out as described above in Section 2.5. As above, we consider the simulation of two methods of sample loading: single and multiple sample solution loading.

#### 3.3.4. Modeling of CLR CCC Separations with Single Sample Solution Loading

To simulate possible options for separating a given mixture of compounds, the following equation can be used [141]:

(23) $$X_{n}(t)=\sum_{i=1}^{n}\frac{1}{\sqrt{\frac{2\pi }{Na^{2}}+\frac{\pi t_{s}^{2}}{6}+\frac{2\pi (i-1)}{N_{ef}a^{2}}}}\exp\left[-\frac{{\left(\frac{i}{a}+\frac{t_{s}}{2}+b(i-1)-t\right)}^{2}}{\frac{2}{Na^{2}}+\frac{t_{s}^{2}}{6}+\frac{2(i-1)}{N_{ef}a^{2}}}\right]$$

$$N_{ef}=\frac{NN_{ec}}{N_{ec}+Na^{2}b^{2}}$$

where *t_s_ =* *τ_s_F/V_c_* is the normalized sample solution loading time.

Equation (23) describes the transformation of the elution profile of the compound *K_D_* at the outlet of the column during the recycling from the first to the last cycle of the separation process *n*.

#### 3.3.5. Modeling of CLR CCC Separations with Multiple Sample Solution Loading

The sample solution is continuously introduced at the inlet of the CCC device over a definite time at the same rate as the mobile phase. The sample solution is repeatedly introduced, when the circulating band of the target compound passes the inlet of the CCC device. The following equations can be used to simulate these separation processes [142]:

(24) $$X_{n}(t)=\frac{1}{\sqrt{2\pi }}\sum_{i=1}^{n-m}\frac{1}{\sigma (i)}\exp\left[-\frac{{\left(t_{R}(i)+t_{Rt}-t\right)}^{2}}{2\sigma^{2}(i)}\right]$$

(25) $$X_{rc}(t)=X_{n,1}(t)+X_{n,2}(t)+\ldots ..+X_{n,ml}(t)$$

$$t_{Rt}=\left(\frac{1}{a_{t}}+\frac{t_{s}}{2}+b\right)m;t_{R}(i)=\frac{i}{a}+\frac{t_{s}}{2}+b(i-1);a_{t}=\frac{1}{1-S+SK_{Dt}};\;\sigma^{2}(i)=\frac{1}{Na^{2}}+\frac{t_{s}^{2}}{12}+\frac{i-1}{N_{ef}a^{2}}$$

where *m* is the number of the cycle, after which the sample solution is re-loaded; *X_n,1_(t), X_n,2_(t),* and *X_n,ml_(t)* are determined by Equation (24); the subscripts 1, 2, and *ml* denote the numbers of the sample solution loadings. The cycle numbers correspond to the numbers of passages of the target compound *K_Dt_* through the CCC device. Counting of time and cycles is carried out from the moment of the first loading of the sample solution.

Equation (24) describes elution profiles of the compound *K_D_* corresponding to individual loading of the sample solution. Equation (25) describes the resulting concentration profiles after several loadings.

Using Equations (24) and (25), the simultaneous separation and concentration of a target compound from a multicomponent sample solution can be simulated to select a suitable process scenarios for a given separation task.

### 3.4. Modeling and Design of Continuous Steady-State Preparative and Industrial Scale Countercurrent Chromatography Separations

The steady-state (SS) regime is the most promising for industrial applications; it provides both high productivity and solvent saving. The SS CCC setup includes two mobile phase tanks—one with the mobile phase and the second with the sample solution in the mobile phase; the mobile phase pump is periodically switched from one tank to another; the sample solution is continuously loaded into the column over a constant time with the constant volumetric rate equal to the flow rate of the mobile phase. The sample solution loading time *τ_s_* (*t_s_*) and the interval between consecutive loads *τ_in_* (*t_in_*) are the free operating parameters of the SS CCC separation processes. The productivity and the separation efficiency are interconnected, and increasing the productivity can lead to the decrease in the purity of the separated products: for maximum performance and minimum solvent consumption, the interval between two consecutive loads of the sample solution *τ_in_* (*t_in_*) must be minimal but sufficient to ensure separation of the adjacent sample bands; on the other hand, for maximum performance, the duration of the loading periods *τ_s_* (*t_s_*) should be as long as possible, but it should not reduce the separation. To find the trade-off between product quality and process performance requires prior mathematical modeling to determine the optimal values of the sample solution loading time and the interval between consecutive loads. To simulate SS CCC separations, it is sufficient to have the theoretical description of the elution profiles after two consecutive sample solution loads.

### 3.5. Conventional Steady-State Countercurrent Chromatography (SS CCC) Separations

The elution profiles of the compound *K_Dj_*, corresponding to the first and second consecutive sample solution loads and the resulting concentration profiles after two loads, can be calculated by the following equations [141,142]:

(26) $$X_{j1}(t)=\frac{1}{\sqrt{\frac{2\pi }{N_{j}a_{j}^{2}}+\frac{\pi t_{s}^{2}}{6}}}\exp\left[-\frac{{\left(\frac{1}{a_{j}}+\frac{t_{s}}{2}-t\right)}^{2}}{\frac{2}{N_{j}a_{j}^{2}}+\frac{t_{s}^{2}}{6}}\right]$$

(27) $$X_{j2}(t)=\frac{1}{\sqrt{\frac{2\pi }{N_{j}a_{j}^{2}}+\frac{\pi t_{s}^{2}}{6}}}\exp\left[-\frac{{\left(\frac{1}{a_{j}}+\frac{t_{s}}{2}+t_{in}-t\right)}^{2}}{\frac{2}{N_{j}a_{j}^{2}}+\frac{t_{s}^{2}}{6}}\right]$$

(28) $$a_{j}=\frac{1}{1-S_{f}+S_{f}K_{Dj}}$$

(29) $$X_{j}(t)=X_{j1}(t)+X_{j2}(t)$$

where $X_{j1}{=x}_{j1}/{\bar{x}}_{j}$ and $X_{j2}{=x}_{j2}/{\bar{x}}_{j}$ are the normalized concentrations of the first and second band profiles, respectively; ${\bar{x}}_{j}{=Q}_{j}/V_{c}{=x}_{js}{F\tau }_{s}/V_{c}$ is the average concentration of the compound *j* in the column after one sample solution load; *Q_j_ = x_js_Fτ_s_* is the amount of the compound *j* loaded during the sample solution loading time *τ_s_*; *x_js_* is the concentration of the solution *j* in the sample solution; *F* is the volumetric flow rate of the fresh mobile phase and the sample solution; *t_in_ =* *τ_in_F/V_c_*, (*τ*_*in*_) is the interval between consecutive sample solution loads.

### 3.6. Steady-State Multiple Dual Mode Countercurrent Chromatography (SS MDM CCC) Separations

The steady-state MDM CCC differs from the non-steady-state MDM CCC by the constancy of the duration of phase elution steps. The duration of the flow periods of the phases is kept constant for all the cycles. At the beginning of the first step of every cycle, the sample solution is continuously loaded into a CCC column over a constant time, not exceeding the duration of the first step. After a certain number of cycles, the steady-state regime is achieved, where concentrations vary over time during each cycle; however, the concentration profiles of compounds eluted with both phases are repeated in all subsequent cycles. The mathematical description of the SS MDM CCC separations has been developed in [143]. Based on these equations, the calculating machine is developed [133] to calculate the elution profiles and the amounts of compounds eluted from a CCC column with the phases during each step and cycle for both non-steady-state and steady-state separations. Examples of the simulation of SS MDM CCC separations using the calculating machine can be found in [133].

### 3.7. Steady-State Closed-Loop Recycling Countercurrent Chromatography (SS CLR CCC) Separations

As in the cases of SS CCC and SS MDM CCC separations, the sample solution at specified intervals ***τ_in_*** (***t_in_***) is continuously loaded into a CCC device over a constant time ***τ_s_*** (***t_s_***). The first loading starts at τ = 0 (*t* = 0). Obviously, the loop must be open during loading the sample solution into the column. After the first loading is finished, the loop is closed, and the solution of compounds circulates in the system until the desired degree of separation is achieved. After that, the loop is opened again; mobile phase is pumped into the column, and the elution of the separated fractions of compounds starts; at τ = *τ_in_* (*t* = *t_in_*), the second portion of the sample solution is continuously loaded into the column over the time ***τ_s_*** (***t_s_***); after the second loading is finished, the mobile phase is pumped into the column until the elution of the compounds of the first load is completed. After that, the loop is closed again, and the second portion of sample solution circulates in the system until the desired separation of compounds is achieved. Furthermore, the operations are repeated. To describe the band profiles after two consecutive loads, the following equations can be recommended [144]:

(30) $$X_{j1n}(t)=\sum_{i=1}^{n}\frac{1}{\sqrt{\frac{2\pi }{N_{j}a_{j}^{2}}+\frac{\pi t_{s}^{2}}{6}+\frac{2\pi (i-1)}{N_{efj}a_{j}^{2}}}}\exp\left[-\frac{{\left(\frac{i}{a_{j}}+\frac{t_{s}}{2}+b(i-1)-t\right)}^{2}}{\frac{2}{N_{j}a_{j}^{2}}+\frac{t_{s}^{2}}{6}+\frac{2(i-1)}{N_{efj}a_{j}^{2}}}\right]$$

(31) $$X_{j2n}(t)=\sum_{i=1}^{n}\frac{1}{\sqrt{\frac{2\pi }{N_{j}a_{j}^{2}}+\frac{\pi t_{s}^{2}}{6}+\frac{2\pi (i-1)}{N_{efj}a_{j}^{2}}}}\exp\left[-\frac{{\left(\frac{i}{a_{j}}+\frac{t_{s}}{2}+b(i-1)+t_{in}-t\right)}^{2}}{\frac{2}{N_{j}a_{j}^{2}}+\frac{t_{s}^{2}}{6}+\frac{2(i-1)}{N_{efj}a_{j}^{2}}}\right]$$

(32) $$N_{efj}=\frac{N_{j}N_{ec}}{N_{ec}+N_{j}a_{j}^{2}b^{2}}$$

(33) $$X_{jn}(t)=X_{j1n}(t)+X_{j2n}(t)$$

where *a_j_* and *N_efj_* are the parameters defined by Equations (28) and (32), respectively; *n* is the number of cycles (the number of passages of the component *j* through the column) required to achieve the desired separation.

Thus, the continuous SS CLR CCC separation is carried out in three repetitive operating steps: (1)—sample solution loading; (2)—separation of compounds in recycling closed-loop; (3)—elution of the separated compounds with the mobile phase. Depending on the composition of the mixture to be separated, the second step can be carried out in several separation stages: after the loading of the sample solution is finished, the loop is closed, and the first separation stage starts; after a certain number of cycles, the first separated compounds are eluted, the loop is closed, and the second separation stage starts; after a number of cycles, other separated compounds are eluted, and so on.

Several examples of simulation and design of SS CLR CCC separations are presented in “Mathcad” software in [144].

Based on the equations presented in this review and the mentioned computer programs, various embodiments of CCC operating modes can be designed and simulated. These equations and computer programs allow determining the purity and productivity of CCC separations under consideration using the necessary experimental information (the values of process parameters *K_D_*, *N*, *S*, etc.) obtained on the available CCC instrument with the selected solvent system.

## 4. Conclusions and Future Work

Countercurrent chromatography is a technology for analytical and preparative-scale separations; it may also be carried out on an industrial scale if semi-continuous sample loading and suitable equipment are used. CCC exhibits high process flexibility and possesses a variety of operating modes unique to the technology, such as MDM CCC and CLR DM CCC. The selection of the operating mode depends on the specific separation task and the available CCC device. Classical mode variations of multiple sample injections can be used to increase the throughput. If the CCC device is not efficient enough to separate a given mixture of substances, then CLR CCC mode separations are best suited to improve the separation. This separation modes are the simplest and most easily implemented ones and do not require significant reconstruction of the experimental setup. To isolate concentrated fractions of target compounds from multicomponent mixtures, the non-steady-state MDM CCC and CLR DM CCC with multiple injections of a sample can be used. Closely related compounds with low partition coefficients can be separated by CLR CCC using recycling systems with long recycling lines. For the separation of closely related compounds with high partition coefficients and the separation of complex mixtures with widely different partition coefficients, the CLR DM CCC mode separations can be recommended. When high productivity is required, continuous steady-state countercurrent chromatography separations are most suitable.

CCC technology is in constant development: new operating schemes and devices are being developed and implemented. Further development of the CCC separation methods considered in this review can be aimed at the following:

Isolation of concentrated fractions of compounds based on the multiple (intermittent) sample loading technique.

Industrial-scale closed-loop recycling dual mode countercurrent chromatography separations based on the semi-continuous sample loading technique.

Closed-loop recycling multiple dual mode countercurrent chromatography separations.
